# Imported Furuncular Myiasis Due to *Cordylobia anthropophaga*: A Case Highlighting the Role of Ultrasonography and Morphological Confirmation

**DOI:** 10.3390/pathogens15080844

**Published:** 2026-08-13

**Authors:** Justyna Adamczuk, Katarzyna Sikorska, Piotr Czupryna, Karol Borawski, Mateusz Matwiejuk, Hend Malla, Merry Jebessa, Anna Moniuszko-Malinowska

**Affiliations:** 1 Department of Infectious Diseases and Neuroinfection, Medical University of Bialystok, Żurawia 14 D, 15-540 Bialystok, Poland; 2 Institute of Maritime and Tropical Medicine, Medical University of Gdansk, Powstania Styczniowego 9b, 81-519 Gdynia, Poland; 3 Department of Dermatology and Venerology, Medical University of Bialystok, Żurawia 14 C, 15-540 Bialystok, Poland; 4 2nd Clinical Department of General and Gastroenterological Surgery, Medical University of Bialystok, 15-540 Bialystok, Poland; 5 Faculty of Medicine, Medical University of Bialystok, Jana Kilińskiego 1, 15-089 Bialystok, Poland

**Keywords:** *Cordylobia anthropophaga*, furuncular myiasis, cutaneous myiasis, travel medicine

## Abstract

Background: Furuncular cutaneous myiasis caused by *Cordylobia anthropophaga* is a rare condition in non-endemic regions and may mimic furunculosis or skin abscesses, posing a diagnostic challenge. Case presentation: A previously healthy 24-year-old woman presented eight days after returning from Zanzibar with multiple tender erythematous nodules on the buttocks and left forearm. One larva was self-extracted, and others were removed under medical supervision. Ultrasound showed subcutaneous tracts consistent with larval infestation. Morphological analysis confirmed *C. anthropophaga*. The patient was treated with extraction and topical therapy. Lesions gradually resolved, leaving minimal residual marks after three months. Conclusions: Clinicians should consider furuncular myiasis in travelers returning from sub-Saharan Africa who present with boil-like nodules. Early recognition and extraction of larvae prevent complications. Traveler education on preventive measures remains essential.

## 1. Introduction

Myiasis is the infestation of human or animal tissues by dipteran larvae. Furuncular myiasis is an important differential diagnosis of persistent boil-like skin lesions in travelers returning from tropical and subtropical regions, where it may be mistaken for bacterial furunculosis, abscesses, or arthropod bites [1,2]. *Cordylobia anthropophaga* (the tumbu fly) is the most common cause of furuncular myiasis in sub-Saharan Africa. Humans become infested after contact with larvae deposited on contaminated clothing, bedding, or soil [1,3,4]. Owing to increasing international travel, imported cases are being reported with increasing frequency in non-endemic countries, where delayed diagnosis remains common because of limited clinician familiarity with the disease [3,4,5].

We report a case of imported furuncular myiasis caused by *C. anthropophaga* in a traveler returning from Zanzibar, highlighting the diagnostic value of ultrasonography, entomological confirmation, and the challenges associated with the management of multiple lesions.

## 2. Case Presentation

A 24-year-old previously healthy woman presented 8 days after returning from Zanzibar with multiple painful erythematous nodules on the buttocks and left forearm. Initially resembling insect bites, the lesions progressed to firm nodules with central openings and visible larval movement. Although the patient did not recall a specific exposure, she had stayed in Zanzibar, where she was exposed to local environmental conditions compatible with *Cordylobia anthropophaga* infestation. The exact route of transmission could not be established retrospectively. One larva was self-extracted, and a few others were removed during admission to the Clinic. In total, five lesions were identified: three on the right buttock, one on the left buttock, and one on the left forearm (Figure 1).

Routine laboratory investigations were unremarkable except for a mildly elevated C-reactive protein concentration. Bacterial cultures obtained from the lesions showed no growth. Ultrasound revealed subcutaneous inflammation and hypoechoic dermal tracts with intralesional movement, suggesting viable parasites (Figure 2). Follow-up revision under ultrasound guidance revealed no larvae (Figure 3). Topical aluminum acetate was initiated.

A follow-up ultrasound two days later showed persistent inflammatory changes. The dominant lesion measured 13 × 12 mm and contained a hypoechoic canal without a visible foreign body, consistent with a resolving post-larval tract. Other tracts were minimal or no longer detectable.

Extracted larvae (Figure 2) were sent to the Institute of Maritime and Tropical Medicine, Medical University of Gdańsk, where morphological examination confirmed *Cordylobia anthropophaga*. Despite gradual improvement, some lesions persisted, and additional larvae were removed during outpatient visits.

During follow-up after 1 month, lesions regressed, leaving only faint residual marks. After dermatological consultation, topical Bedicort G was prescribed for post-inflammatory changes. No signs of active myiasis or secondary infection were observed (Figure 4).

At final follow-up after 2 months, lesions had nearly completely resolved with minimal scarring (Figure 5). The patient reported intermittent numbness and burning in the gluteal region. Further evaluation, including repeat ultrasound and spinal MRI, was planned to assess possible neuropathic sequelae. No evidence of persistent myiasis was found.

## 3. Discussion

Imported furuncular myiasis caused by *Cordylobia anthropophaga* remains an uncommon diagnosis in Europe despite increasing international travel to endemic regions. Because the initial presentation closely resembles bacterial furunculosis, inflamed epidermoid cysts, or arthropod bites, diagnosis is frequently delayed, resulting in unnecessary antibiotic treatment or surgical intervention [6,7,8,9].

Our patient presented with multiple painful nodules that initially mimicked inflammatory skin lesions. The diagnosis became more evident after visible larval movement was observed and was further supported by ultrasonography. In our case, ultrasound proved particularly useful because it demonstrated characteristic hypoechoic dermal tracts containing moving intralesional structures compatible with viable larvae. Moreover, follow-up ultrasound allowed differentiation between persistent inflammatory tracts and ongoing infestation after larval removal. These findings support previous reports indicating that ultrasonography is a valuable adjunctive tool for both diagnosis and treatment monitoring in furuncular myiasis [5,10,11,12,13].

Although the clinical appearance was highly suggestive of furuncular myiasis, entomological confirmation remained important. Morphological identification of *C. anthropophaga* established the diagnosis, confirmed the geographical origin of infection, and contributed to epidemiological documentation of an imported case. Such confirmation is particularly valuable in non-endemic countries, where clinicians and laboratories have limited experience with tropical ectoparasitoses [14,15].

Mechanical extraction remains the cornerstone of treatment for furuncular myiasis. In our patient, repeated extraction procedures resulted in gradual clinical improvement [5,16,17].

An additional feature of our case was the persistence of intermittent numbness and burning sensations after complete larval removal. Although these symptoms gradually improved and no evidence of persistent infestation was found, they most likely reflected post-inflammatory irritation of superficial sensory nerves rather than ongoing parasitic infection. Similar transient neuropathic symptoms have occasionally been described in previous reports [18].

Recently, Biernat et al. reported imported cases of furuncular myiasis caused by *C. anthropophaga* and *C. rodhaini* in Polish travellers returning from sub-Saharan Africa. Similar to our patient, the diagnosis was established following travel to an endemic region and confirmed by morphological examination of the extracted larvae. Their findings emphasize that imported furuncular myiasis remains an uncommon but increasingly recognized travel-associated disease in Europe and may initially be mistaken for bacterial skin infections. Our case further supports the importance of including furuncular myiasis in the differential diagnosis of painful nodular skin lesions in returning travellers and highlights the additional value of ultrasonography in the diagnostic work-up prior to larval extraction [19].

## 4. Conclusions

This case highlights the importance of considering imported furuncular myiasis in travellers returning from sub-Saharan Africa who present with furuncle-like skin lesions, particularly when the clinical course is atypical or conventional antibacterial therapy is ineffective. Ultrasonography proved to be a useful adjunctive diagnostic tool by demonstrating subcutaneous abnormalities compatible with furuncular myiasis and by documenting regression of the lesions during follow-up. Species identification by a reference entomology laboratory was essential for establishing the final diagnosis and contributes to epidemiological surveillance of imported parasitic diseases. This case adds to the growing number of imported furuncular myiasis cases reported in Europe and underscores the importance of travel medicine awareness, careful clinical evaluation, and multidisciplinary collaboration in the diagnosis and management of uncommon tropical infections in non-endemic settings.

## Figures and Tables

**Figure 1:**
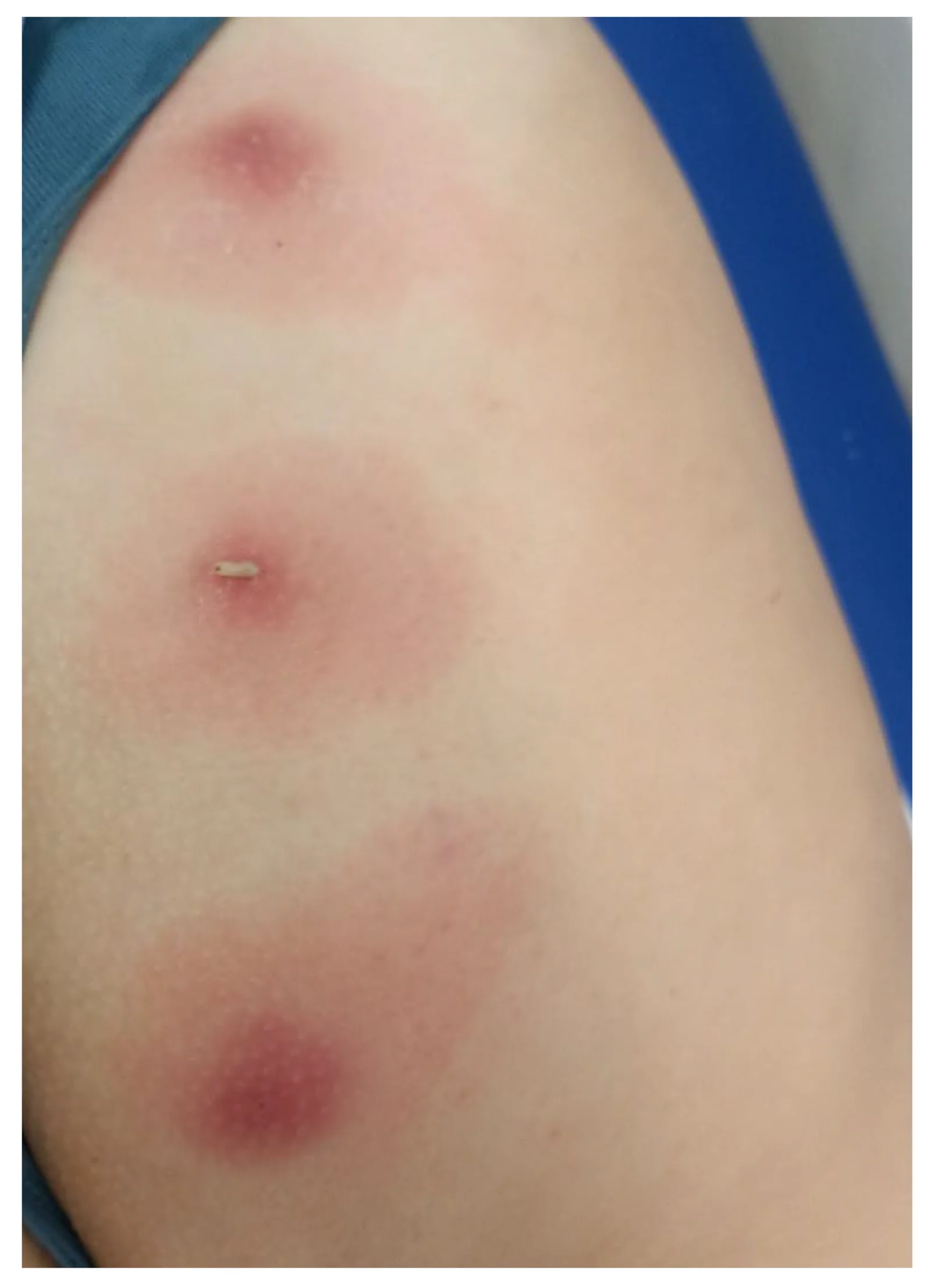
Multiple erythematous inflammatory nodules are visible on the skin; in one lesion, a cream-coloured larva protrudes through a central opening. Photo by J. Adamczuk.

**Figure 2:**
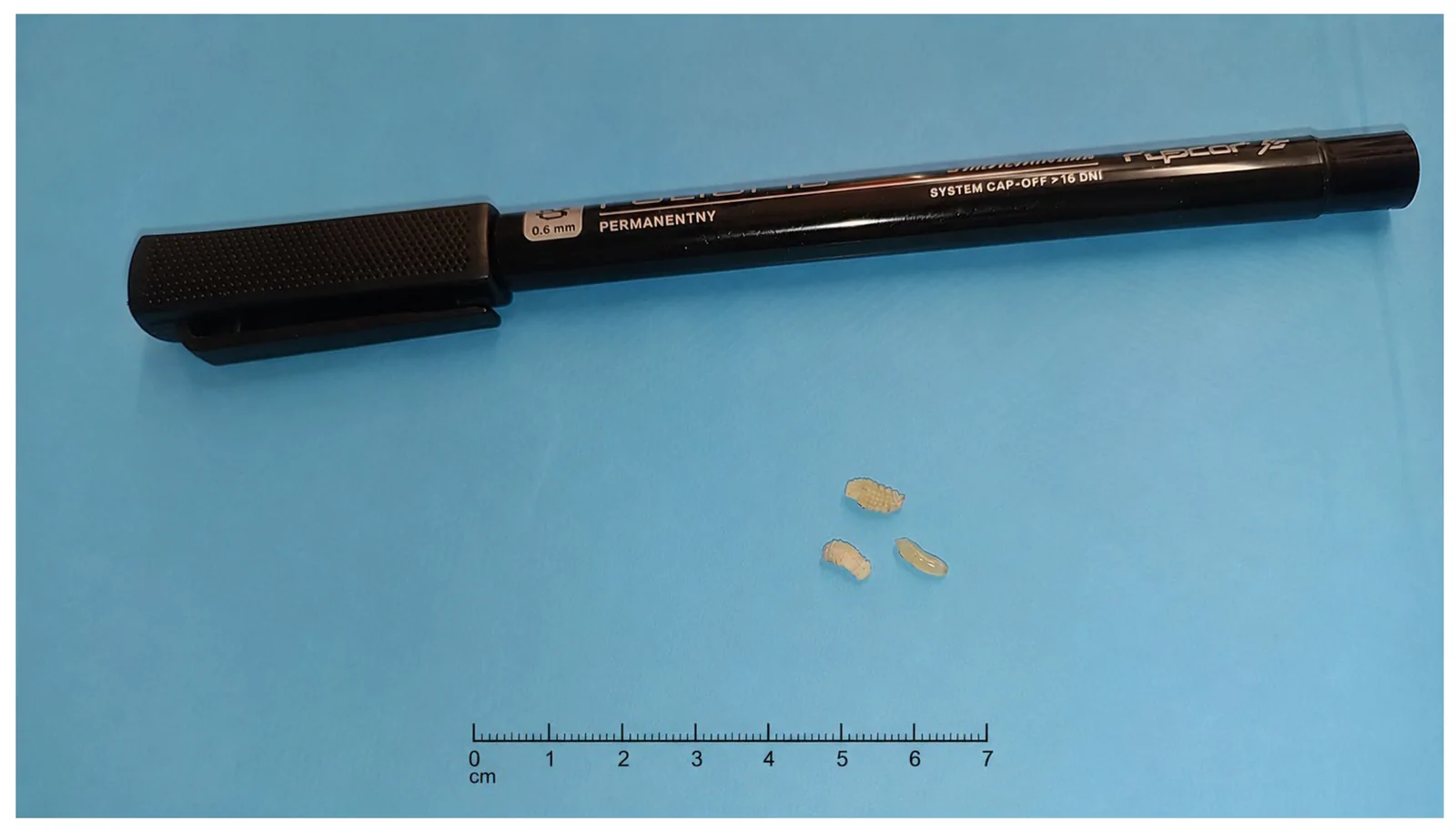

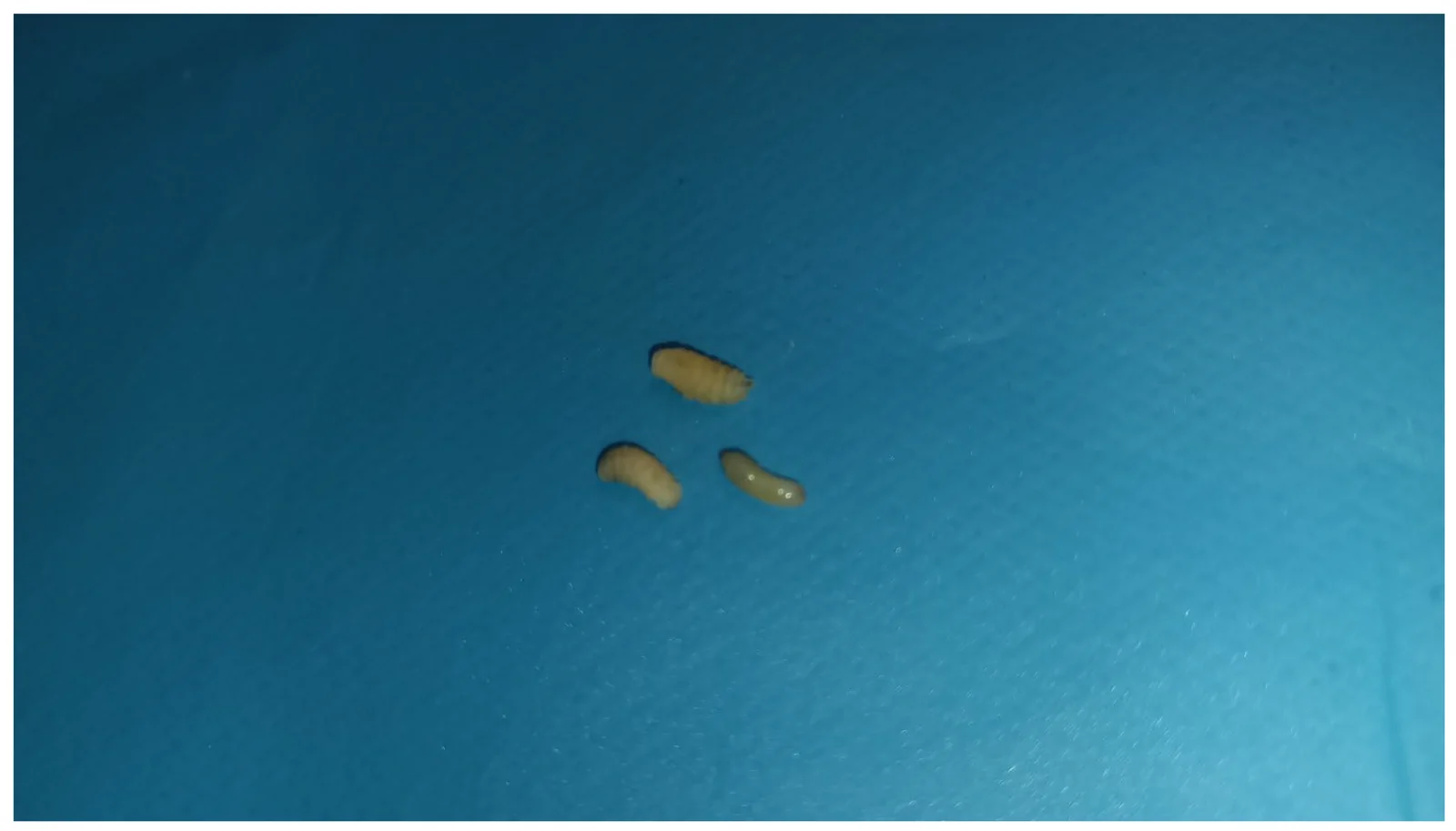
Extracted *Cordylobia anthropophaga* larvae measuring approximately 0.5–1.3 cm in length. Morphological identification was performed at the Institute of Maritime and Tropical Medicine, Medical University of Gdańsk. Identification was based on the characteristic morphology of third-instar larvae, including the robust barrel-shaped body, prominent posteriorly directed cuticular spines arranged in distinct rows, the typical posterior spiracular plates, and the overall body proportions, which are consistent with *Cordylobia anthropophaga*.

**Figure 3:**
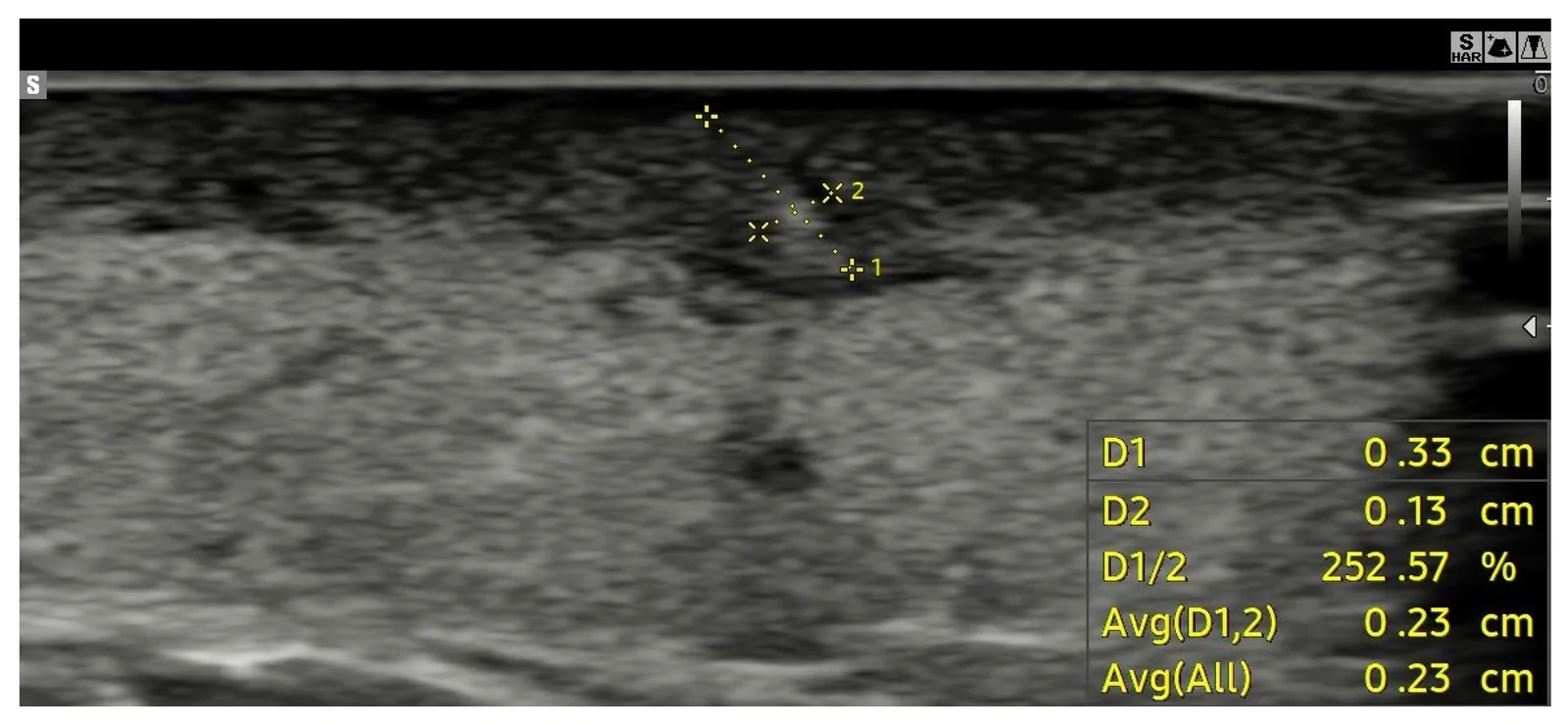
Ultrasound demonstrated a small focal hypoechoic lesion containing a central hyperechoic structure, surrounded by hyperechoic inflammatory changes within the subcutaneous tissue, compatible with furuncular myiasis. The yellow marks represent measurement calipers automatically generated by the ultrasound device during image acquisition.

**Figure 4:**
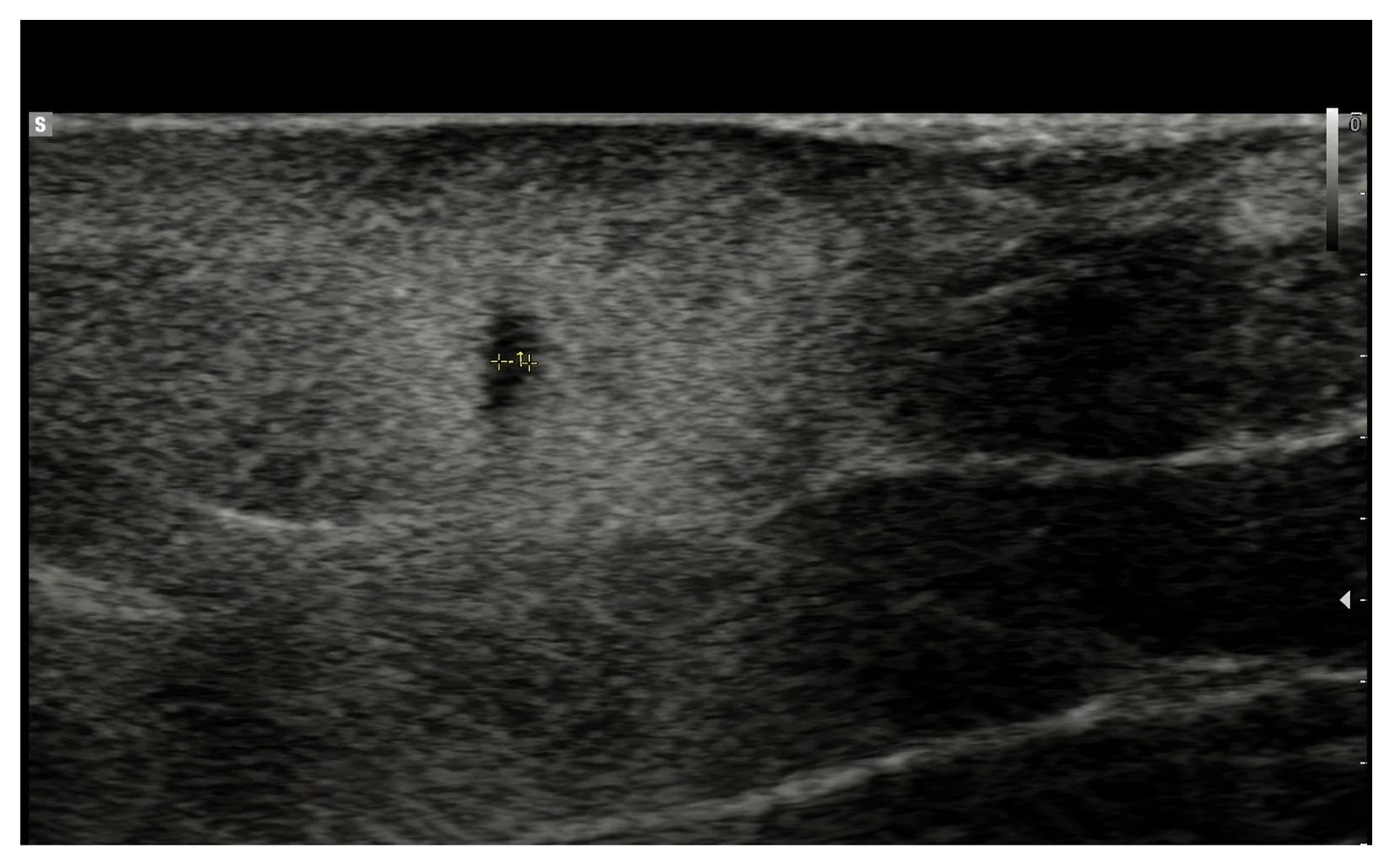
Follow-up ultrasonographic examination performed after larval extrusion. Marked reduction in the lesion was observed, with only a tiny residual hypoechoic tract remaining within the subcutaneous tissue, consistent with post-inflammatory changes.

**Figure 5:**
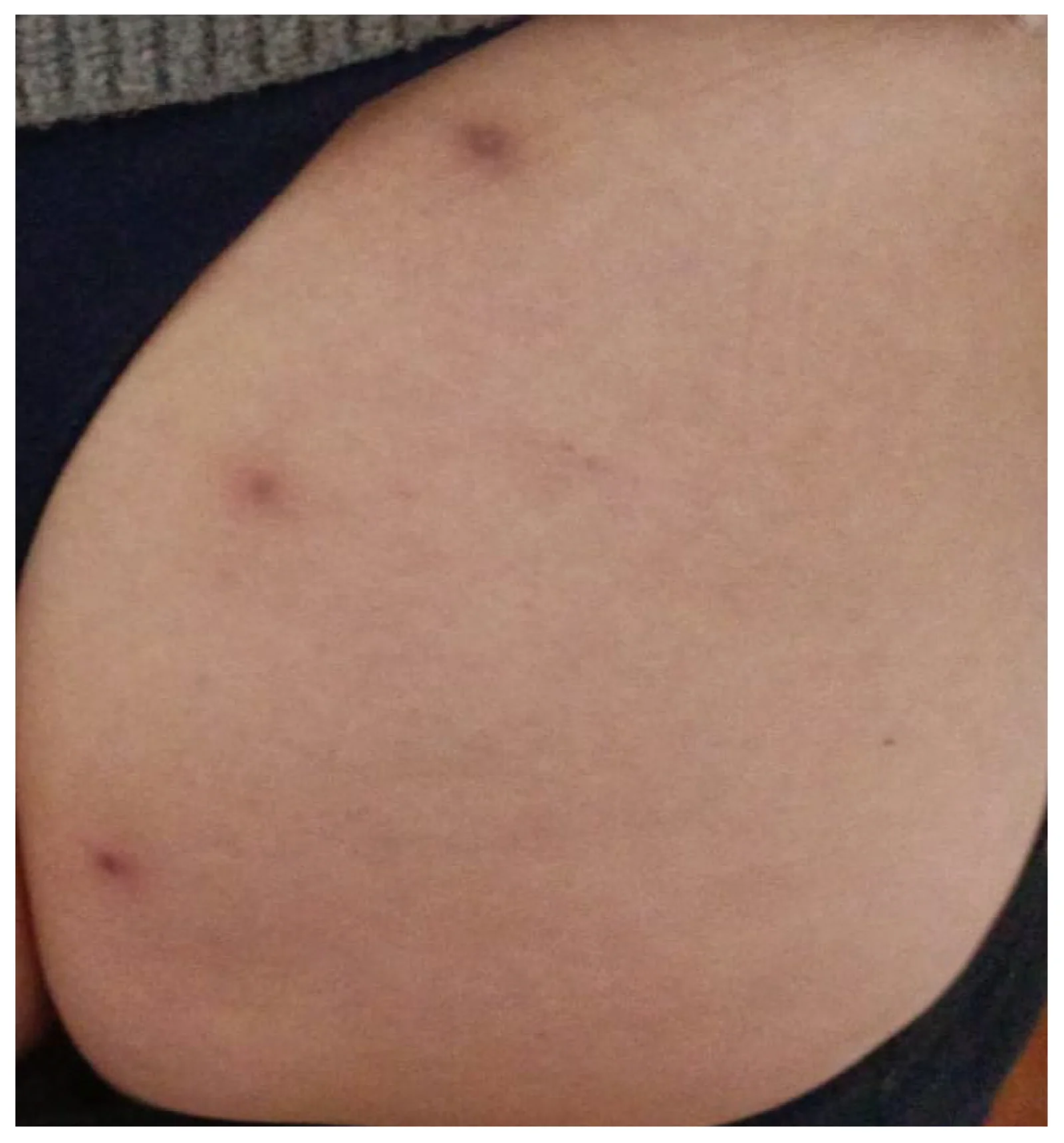
Follow-up photograph of the lesion on the patient’s buttock after treatment, demonstrating progressive healing, with no palpable pathological masses.

## Data Availability

Data supporting the findings of this study are available within the article. Additional data are available from the corresponding author upon reasonable request, subject to patient confidentiality requirements.
